# The use of alpha-1 adrenergic blockers in children with distal ureterolithiasis: a systematic review and meta-analysis

**DOI:** 10.1590/S1677-5538.IBJU.2015.0048

**Published:** 2015

**Authors:** F.P. Glina, P.M.V. Castro, G.G.R. Monteiro, G.C. Del Guerra, S. Glina, M. Mazzurana, W.M. Bernardo

**Affiliations:** 1 Faculdade de Ciências Médicas de Santos, Centro Universitário Lusíada, Santos, São Paulo, Brasil;; 2 Departamento de Urologia da Faculdade de Medicina do ABC, Santo André, São Paulo, Brasil;; 3 Departamento de Cirurgia Geral do Hospital Guilherme Álvaro, Santos, São Paulo, Brasil;; 4 Associação Médica Brasileira, São Paulo, Brasil

**Keywords:** Ureterolithiasis, Adrenergic alpha-1 Receptor Antagonists, Child, Review Literature as Topic

## Abstract

**Introduction:**

Urinary lithiasis is the main urologic cause of emergency treatment in adult patient. In the past years, the incidence in children population has increased. However, literature about the use of alpha-1 adrenergic blockers in pediatric population with distal ureterolithiasis is still scarce. The drug acts by decreasing ureter contractions, especially in the distal portion, facilitating calculus expulsion.

**Objective:**

This review has the objective to evaluate the use of alpha-1 adrenergic blockers as medical expulsive treatment in children with distal ureterolithiasis.

**Evidence Acquisition:**

An electronic literature search was performed using the MEDLINE, COCHRANE, and LILACS databases. We further searched manually the references of the primary studies. Searches were concluded on October 4th, 2014. Articles were selected, independently and in pairs, by the respective titles and summaries. Any divergence was resolved by consensus.

**Evidence Synthesis:**

Alpha-1 adrenergic antagonists increased the probability of calculus expulsion by 27% (NNT=4). Calculi smaller than 5mm, increased by 33% (NNT=3). Larger than 5mm, increased by 34% (NNT=3).

**Conclusion:**

Alpha-1 adrenergic blocker use is related with a greater incidence of expulsion of ureteral calculi, smaller or greater than 5mm, and fewer episodes of pain when compared to ibuprofen. However it is necessary larger samples to enhance the power analysis of the expulsion of ureteral calculi larger than 5mm and the episodes of pain.

**Patient Summary:**

This review analyzed the outcome of alpha adrenergic antagonist in children with ureteral calculi. We conclude that it is the best medicine for use, since it helps the expulsion of the stone.

## INTRODUCTION

Urinary lithiasis is the main urologic cause of emergency treatment in adult patients (1). It can occur at any age, including children.

In the United States, during the 1950s, the disease was the cause for hospitalization in one out of every 7600 pediatric patients; in the 1990s, in one out of every 1000, and between 2002 and 2007, in one out of every 685 (2, 3). The reason for this increased incidence is not clear. There are controversial theories that associate these numbers to eating and life habits.

Typical in the pediatric patient, ureterolithiasis consists of a calcium oxalate calculus, found in 55% of cases in the distal ureter (4). The clinical picture consists of general symptoms, such as unspecific pain in the abdomen, flanks, or pelvis (5). Additionally, 90% of the cases manifest with macroscopic or microscopic hematuria, and may progress with acute urinary tract infections and urinary retention.

The prevalence of cases increased with areas that are hot, arid, and have dry climate. Therefore, the locations most affected are United States, British Isles, Scandinavian countries, Central Europe, Mediterranean countries, Turkey, Pakistan, north of India, parts of the Himalayan Peninsula, China, north of Australia (6, 7).

Treatment is determined by the size of the calculus and clinical picture. Those smaller than 5mm are generally eliminated without intervention, whereas the largest stones are commonly treated by extracorporeal lithotripsy, ureteroscopy, and percutaneous nephrolithotomy (6, 8). The presence of urinary infection is an indication for surgical intervention, regardless of the size of the calculus.

In cases where there is no associated urinary infection and pain is not intense or is controlled with analgesics, a “wait-and-see” approach may be taken, expecting the spontaneous elimination of the calculus.

In the adult patient, there are various conservative treatments to treat calculi smaller than 12mm, such as the use of calcium blockers, non-steroidal anti-inflammatory agents, or alpha-1 adrenergic blockers, which is the better approach (9). The blocker acts by decreasing ureter contractions, especially in the distal portion, facilitating calculus expulsion. However, in the pediatric population, literature is still scarce (3, 10). There are papers with high strength of evidence, but with few cases.

## OBJECTIVE

This review has the objective to evaluate the use of alpha-1 adrenergic blockers as medical expulsive treatment in children with distal ureterolithiasis.

## EVIDENCE ACQUISITION

### 

#### Identification and selection of studies

An electronic literature search was performed using the MEDLINE, COCHRANE, and LILACS databases.

The MEDLINE research was made through PubMed using the combination of the terms (Ureteral Calculi OR ureteral stone) AND (Adrenergic alpha-Antagonists) AND (Child* OR Adolescent). At LILACS, the following search strategy was used: strategy (alpha adrenergic antagonist) AND filters (Therapy and Children and Adolescent). At COCHRANE database, the strategy was (Adrenergic alpha antagonists) AND (Child). We further searched manually through the references of the primary studies. The searches were concluded on October 4^th^, 2014.

The articles were selected, independently and in pairs, by reading the respective titles and summaries. Any divergence was resolved by consensus.

#### Inclusion and exclusion criteria

The inclusion criteria used consisted of the following: randomized clinical trials comparing the use of an alpha-1 adrenergic antagonist to standard analgesia in children with distal ureterolithiasis.

The exclusion criteria covered non-randomized clinical trials, cohort and case-control studies, patients with proximal ureterolithiasis and papers about adult population.

#### Outcomes analyzed

The outcomes analyzed were calculus expulsion, pain episodes (as necessity of analgesia and hospitalizations), expulsion of calculi smaller than 5mm and expulsion of calculi larger than 5mm.

#### Methodological Quality

The methodological quality of the primary studies was evaluated by the GRADE system proposed by the Grades of Recommendation, Assessment, Development and Evaluation group (11). The system offers several advantages in comparison to other evidence grading systems. One important advantage is to separate the quality evaluation of the evidence from the strength of recommendation evidence.

### Statistical Analysis

The meta-analysis was performed with the Cochrane Review Manager 5.2 program (12). Data were evaluated by intention-to-treat.

The evaluation of the dichotomic variables was performed by the difference in absolute risk adopting a 95% confidence interval. When there was a statistically significant difference between the groups, the number needed to treat (NNT) or the number needed to cause harm (NNH) was calculated.

The continuous variables were evaluated by the difference in means. Studies that did not show data in terms of means and their respective standard deviations were not included in the analyses.

The power of analysis was calculated using the program Open Epi 3.03 (13). It was considered statistically relevant power greater than 80%.

#### Heterogeneity and sensitivity analysis

Inconsistencies among the clinical studies were estimated using the chi-squared heterogeneity test and quantified using I^2^. A value above 50% was considered substantial. Studies that generated heterogeneity were represented by funnel plots.

## EVIDENCE SYNTHESIS

### 

#### Selection of studies

**Figure 1:**
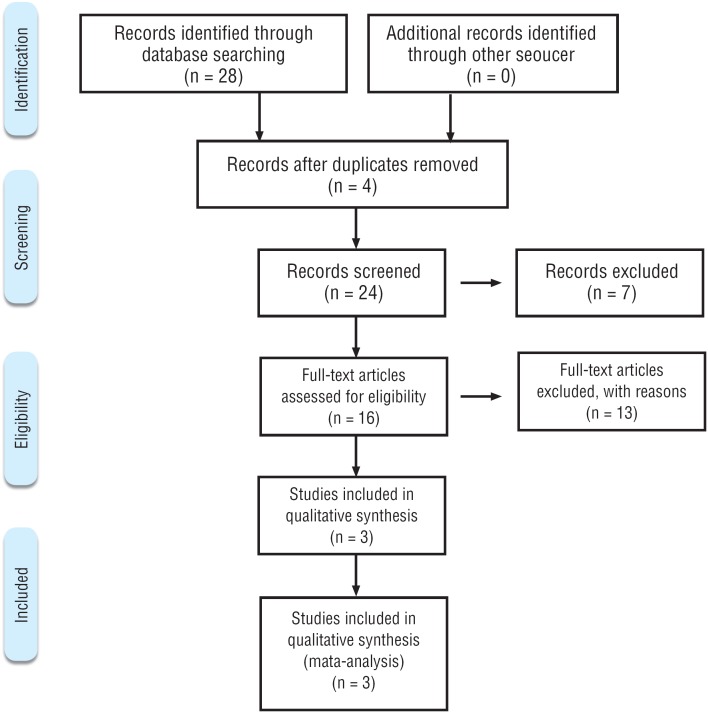
Prisma 2009 Flow Diagram (15).

A total of 28 articles (MEDLINE=23; COCHRANE=3; and LILACS=2) were retrieved by electronic searches. In the manual search no articles were found in addition to those previously selected. Three articles were found both in MEDLINE and in COCHRANE and one article was found both in MEDLINE and in LILACS; three were excluded by the title, seven by the reading of the abstract because they were not in English or not about distal ureterolithiasis. Ten other articles were excluded after full reading the papers: one for being a cohort, two for being a review and seven for not dealing with pediatric patients. Thus, three randomized clinical trials were preselected and included in this review (Figure-1).

The three studies included patients randomized into two groups, totaling up 145 patients; 76 were in the intervention group (alpha-1 adrenergic antagonist) and 69 in the control group (ibuprofen).

Methodological quality evaluation performed by GRADE is represented on Table-1.

**Table 1:** Methodological evaluation by GRADE.

PARAMETERS	AYDOGDU 2009	MOKHLESS 2012	ERTURHAN 2013
Whas the study randomized	Y	Y	Y
Was the allocation of patients to the groups confi dential?	Y	Y	Y
Were the patients analyzed in the groups to which they were randomized (was the analysis by intention-to-treat)?	Y	Y	Y
Were the patients analyzed in the groups to wich they were known prognostic factors?	Y	Y	Y
Was the study blind?	ND	ND	ND
Except for the experimental intervention, were the groups treated equally?	Y	Y	Y
Were the losses signifi cant?	N	N	N
Did study have a precision estimate for the effects of treatment?	Y	N	N
Are the study patients similar to those of interest?	Y	Y	Y
Are the outcomes of the study clinically relevant?	Y	Y	Y
Were the potential confl icts of interest declared?	N	N	N

Legend: Y: Yes, N: No, ND: Not Described.

#### Description of the studies included

The study by Aydogdu et al. (10) consisted of a prospective study and included 39 patients with distal ureterolithiasis. These patients were randomized into two groups, 19 in the group of alpha-1 adrenergic antagonists and 20 in the ibuprofen group. The rate of calculus expulsion, mean time for expulsion, and adverse events of alpha-1 adrenergic antagonists are the outcomes evaluated.

In the study by Erturhan et al. (15), 45 patients with distal ureterolithiasis were randomized; 24 in the alpha-1 adrenergic antagonist group and 21 in the ibuprofen group. The outcomes analyzed were the rate of calculus expulsion and the mean time of expulsion. Median number of pain episodes was 1 (interquartile range 1-1) in the alpha-1 adrenergic antagonist group and 1 (interquartile range 1-2) in the ibuprofen group (p=0.023).

The study by Mokhless et al. (17) is a randomized prospective study, carried out between 2007 and 2010, which analyzed 61 patients, 33 in the alpha-1 adrenergic antagonist group and 28 in the ibuprofen group. The rate of calculus expulsion, mean time of expulsion, the need for analgesia, and possible adverse effects of the drugs were evaluated. Number of pain episodes was 1.4±1.2 (Mean±SD) in the alpha-1 adrenergic antagonist group and 2.2±1.4 in the ibuprofen group (p<0.02) (Table-2).

**Table 2:** Description of the included studies.

Author	Number of Patients AA1A	Number of Patients SA	Age of Children (Years)		Treatment		Stone Passage Mesured by
			AA1A+SA	SA	**AA1A+SA**	**SA**	
Aydogdu (10)	19	20	6.2±2.4	5.1 ±2.2	**Doxazosin** 0.03mg/Kg/day **Ibuprofen** 10mg/Kg 2x/day	**Ibuprofen** l0mg/Kg2-4x/ day	Urinary filtration
Erturhan (15)	24	21	6.0±3.5	7.2±3.5	**Doxazosin** 0.03mg/Kg/day **Ibuprofen** 10mg/Kg 2-4x/day	**Ibuprofen** 10mg/Kg 2-4x/ day	X-Ray KUB and US and NCCT*
Mokhless(16)	33	28	7.3.0±4.2	7.1 ±3.2	**Tamsulosin** >4years:0.4mg <4years:0.1mg **Ibuprofen** ND	**Placebo** ND **Ibuprofen** ND	Urinary filtration

Legend: AA1A: alpha-1 adrenergic antagonist; SA: standard analgesia; ND: not described; x/d: Times per day; X-Ray KUB: radiography of the kidneys, ureters, and bladder; US: ultrasonography; NCCT: non-contrast-enhanced spiral computed tomography;

* In case of any suspicion.

#### Analysis of Calculus Expulsion

**Figure 2:**
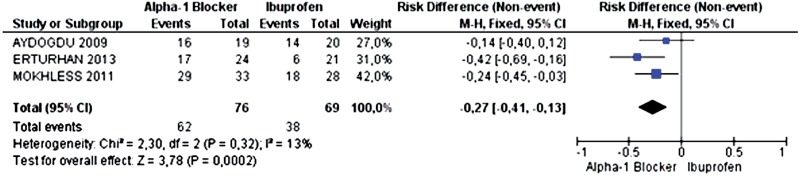
Meta-analysis of the incidence of ureteral calculus expulsion.

The three primary studies analyzed the outcome of calculus expulsion. The incidence of ureteral calculus expulsion was 81.58% in the alpha-1 adrenergic antagonist group (62 out of 76 patients) and 55.07% in the ibuprofen group (38 of 69 patients). The alpha-1 adrenergic antagonists increased the probability of calculus expulsion by 27% (95% CI 0.13 to 0.41; p=0.0002 and I^2^=13%), needing to treat 4 patients to achieve this benefit (NNT=4) (Figure-2). Power was 93.4%.

#### Analysis relative to pain episodes

**Figure 3:**
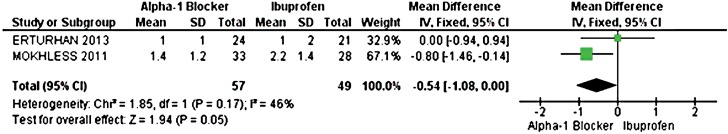
Meta-analysis of the difference in means of pain episodes.

Two primary studies analyzed the pain episode outcome. The difference in the mean between the groups was 0.54 (95% CI 0.00 to 1.08; p=0.05 and I^2^=46%). The alpha-1 adrenergic antagonists decreased the mean of pain episodes (Figure-3). Power was 61.46%.

#### Analysis of the Expulsion of Calculi Smaller than 5mm

**Figure 4:**
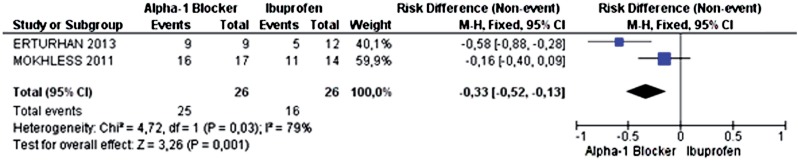
Meta-analysis of the incidence of ureteral calculi expulsion smaller than 5mm.

**Figure 5:**
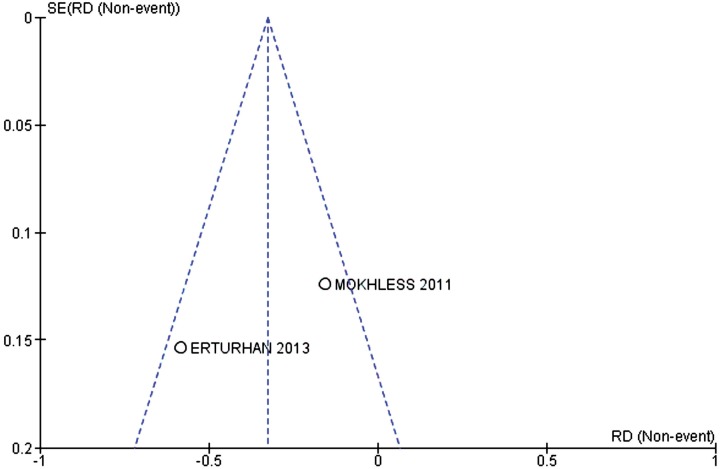
Funnel-plot of the outcome that presented heterogeneity above than 50%.

Two primary studies analyzed the outcome of expulsion of calculi smaller than 5mm. The incidence of ureteral calculus expulsion was 96.15% in the alpha-1 adrenergic antagonist group (25 out of 26 patients) and 61.54% in the ibuprofen group (16 out of 26 patients). The alpha-1 adrenergic antagonists increased the probability of expulsion of calculi smaller than 5mm by 33% (95% CI 0.13 to 0.52; p=0.001) and I^2^=79%), with 3 patients needing treatment to achieve this benefit (NNT=3) (Figure-4). The funnel-plot of this outcome is represented in Figure-5. Power was 88.69%.

#### Analysis of Expulsion of Calculi Larger than 5mm

**Figure 6:**
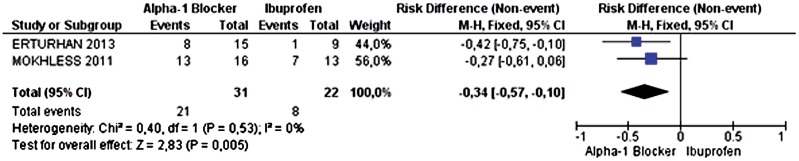
Meta-analysis of the incidence of expulsion of ureteral calculi larger than 5mm.

Two primary studies analyzed the outcome of expulsion of calculi larger than 5mm. The incidence of ureteral calculus expulsion was 67.74% in the alpha-1 adrenergic antagonist group (21 out of 31 patients) and 36.36% in the ibuprofen group (8 out of 22 patients). The alpha-1 adrenergic antagonists increased the probability of calculus expulsion by 34% (95% CI 0.10 to 0.57 p=0.005 and I^2^=0%), with 3 patients needing treatment in order to achieve this benefit (NNT=3) (Figure-6). Power was 62.45%.

## DISCUSSION

Symptomatic ureterolithiasis represents the most frequent urological patients in emergency services (1). Over the last two decades, with the development of extracorporeal lithotripsy associated with the progress of endourology and the appearance of progressively less rigid or even flexible endoscopes, there has been an advance in the treatment of ureterolithiasis (10). Nevertheless, despite such procedures being extremely effective, with success rates between 98.5% and 100%, (15) it is imperative to evaluate the high cost and risk of complications, such as perforation, avulsion, and ureteral narrowing reported in about 3 to 5% of the procedures (1, 10). Thus, the pharmacological treatment seeking the spontaneous expulsion of stones is amply preferred as the first choice of treatment (1).

Even so, the spontaneous expulsion of distal ureteral calculi depends on various factors, including size, number and location, smooth muscle spasm, and ureteral edema (11, 16). In this context, prior studies demonstrated that the inhibition of alpha-1 receptors located primarily in the distal ureteral smooth muscle reduces intra-ureteral pressure and increases peristalsis, therefore favoring calculus elimination (10). Additionally, some studies clearly concluded that the use of alpha-1 adrenergic inhibitors could also be beneficial for residual fragments after extracorporeal shock lithotripsy (15). Increasing the level of evidence of the result of isolated studies, two meta-analyses identified clinical benefit in the use of alpha-blockers in adult patients with ureteral calculi by showing that the use of alpha-blockers compared to placebo increased the probability of calculus expulsion by 52% and 44%, respectively (1).

And finally, despite ureteral calculi having been amply studied in adults, to date the same benefits of alpha blockers have not been confirmed in the pediatric population based on the meta-analysis of randomized clinical trials (10).

The systematic review with meta-analysis is a type of study with scientific precision that selects the best evidence available in medical literature and demonstrates the methodological quality of the primary studies, which is a fundamental condition for attaining precise conclusions on the effect of interventions. To avoid distortions, it was decided to only include results with clinical and statistical homogeneity.

The search strategy showed that there are few controlled and randomized clinical trials available that compare alpha-1 adrenergic antagonists and ibuprofen in the treatment of distal ureterolithiasis in children.

A possible source of bias may be the difference between the processes of randomization of the studies included. However, the quality of the allocation process was considered adequate in all studies. All patients analyzed met the defined eligibility criteria. In the statistical analysis, calculation of the size of the sample and the analysis as per intention-to-treat were used. A common limitation of the analysis of the outcomes was the variety of alpha-1 adrenergic antagonists and their dosages.

The last source of limitation or bias would be the difference of methods for measuring calculi expulsion (Table-2). The study by Erturhan et al. (15) measured the expulsion rate through imaging studies (X-ray, ultrasound and non-contrast CT), method with higher accuracy. The two other studies, Mokhless et al. (16) and Aydogdu et al. (10) analyzed it through urine filtration, a less sensitive method, although with higher specificity. However, we understand that this was not a confounding factor in our analysis. On three analyzes in which this measurement could influence (Figures 2, 4 and 6), the result directly reflected the outcome of the study with more accuracy, Erturhan et al. (15). Then we can infer that, in fact, the improvement generated by alpha blocker is even greater than that was found in the meta-analysis, since two of the studies used as the measuring standard a less consistent method.

To claim that the result of a study with a small sample is statistically significant is required to evaluate the error type 1, when p is less than 5%. This ensures that the result is actually true, as all the analyzes in this meta-analyzes. To state that a study is reproducible is necessary to evaluate the error type 2, when the power is greater than 80%. This ensures that, if the study would be remade elsewhere would have the same result. This is the case for the analysis of calculus and expulsion of calculi smaller than 5mm (Figures 2 and 4). However, studies with power less than 80% requires a larger sample to affirm reproducibility, as in the case of analysis relative to pain episodes and expulsion of calculi larger than 5mm (Figures 3 and 6).

The study followed the ethical and confidentiality principles of information that are recommended, since it is an analysis of results already published in other articles, and the formal approval of a research ethics committee was not necessary.

## CONCLUSIONS

The use of an alpha-1 adrenergic blocker is related with a greater incidence of expulsion of ureteral calculi, smaller or greater than 5mm, and fewer episodes of pain when compared to ibuprofen. However, it is necessary larger samples to enhance the power analysis of the expulsion of ureteral calculi larger than 5mm and the episodes of pain.
